# Antibiotic Prescribing Practices and Errors among Hospitalized Pediatric Patients Suffering from Acute Respiratory Tract Infections: A Multicenter, Cross-Sectional Study in Pakistan

**DOI:** 10.3390/medicina55020044

**Published:** 2019-02-11

**Authors:** Sadia Iftikhar, Muhammad Rehan Sarwar, Anum Saqib, Muhammad Sarfraz, Qurat-ul-ain Shoaib

**Affiliations:** 1 Akhtar Saeed College of Pharmaceutical Sciences, Lahore 54000, Pakistan; thesadiyaiftikhar@gmail.com; 2 Department of Pharmacy, The Islamia University of Bahawalpur, Bahawalpur 63100, Punjab, Pakistan; anumsaqibzaidi@gmail.com (A.S.); muhammad.sarfraz@aau.ac.ae (M.S.); 3 College of Pharmacy, Al Ain University of Science and Technology, Al Ain, PO Box 64141, Abu Dhabi, UAE; 4 College of Pharmacy, University of the Punjab, Lahore 54000, Pakistan; anz.pharmacist@gmail.com

**Keywords:** pediatrics, antibiotics, acute respiratory tract infections, prescribing errors

## Abstract

*Background and objective:* The noncompliance of treatment guidelines by healthcare professionals, along with physiological variations, makes the pediatric population more prone to antibiotic prescribing errors. The present study aims to evaluate the prescribing practices and errors of the most frequently prescribed antibiotics among pediatric patients suffering from acute respiratory tract infections who had different lengths of stay (LOS) in public hospitals. *Methods:* A retrospective, cross-sectional study was conducted in five tertiary-care public hospitals of Lahore, Pakistan, between 1 January 2017 and 30 June 2017. The study population consisted of pediatric inpatients aged 0 to 9 years. *Results:* Among the 11,892 pediatric inpatients, 82.8% were suffering from lower acute respiratory tract infections and had long LOS (53.1%) in hospital. Penicillins (52.4%), cephalosporins (16.8%), and macrolides (8.9%) were the most frequently prescribed antibiotics. Overall, 40.8% of the cases had antibiotic prescribing errors related to wrong dose (19.9%), wrong frequency (18.9%), and duplicate therapy (18.1%). Most of these errors were found in the records of patients who had long LOS in hospital (53.1%). Logistic regression analysis revealed that the odds of prescribing errors were lower in female patients (OR = 0.6, 95% CI = 0.1–0.9, *p*-value = 0.012). Patients who were prescribed with ≥3 antibiotics per prescription (OR = 1.724, 95% CI = 1.1–2.1, *p*-value = 0.020), had long LOS (OR = 12.5, 95% CI = 10.1–17.6, *p*-value < 0.001), and were suffering from upper respiratory tract infections (URTI) (OR = 2.8, 95% CI = 1.7–3.9, *p*-value < 0.001) were more likely to experience prescribing errors. *Conclusion:* Antibiotics were commonly prescribed to patients who had long LOS. Prescribing errors (wrong dose, wrong frequency, and duplicate therapy) were commonly found in cases of lower respiratory tract infections (LRTIs), especially among those who had prolonged stay in hospital.

## 1. Introduction

Acute respiratory tract infections (ARTIs), i.e., upper respiratory tract infections (URTIs) and lower respiratory tract infections (LRTIs), are responsible for increased morbidity and mortality rates among children [1,2]. Children are more prone to ARTIs because of their weak immune system and constant contact with persons who could be carriers [3]. According to an estimation made by the World Health Organization (WHO), ARTIs account for 20% of all the childhood deaths [4]. Globally, 40% of the mortalities due to ARTIs have been reported in developing countries like Nepal, Bangladesh, Indonesia, and India [5]. According to an estimate made by the World Bank collection of development indicators, the prevalence of ARTIs among Pakistani children was found to be 17.7% in 2013. However, only 60.3% of them could get access to treatment [6].

Most of the ARTIs are caused by viruses, but the unnecessary prescribing of antibiotics like penicillins, cephalosporins, and macrolides in children suffering from ARTIs is a common practice globally [7]. In many healthcare settings, the antibiotic prescribing rate is twice as much as the expected rate of antimicrobial needed [8]. Although various international guidelines recommend the limited use of antibiotics in ARTIs, the noncompliance of these guidelines and the frequency of prescribing antibiotics in these infections is common in the healthcare system of Pakistan [9,10]. Evidence suggests that the unwanted use of antibiotics (e.g., cephalosporins) in pneumonia and bronchitis has economically burdened the healthcare system by causing a significant increase in the incidence of antimicrobial resistance, prescribing errors, and patients’ length of stay (LOS) in hospital [11,12].

The term “prescribing errors” can be defined as “the prescribing decision that cause an unintentional and significant reduction in the effectiveness of the treatment or increase in the risk of harm compared to the with the practices mentioned in standard treatment guidelines” [13]. The Infectious Diseases Society of Pakistan (IDSP) guidelines for antibiotic use are available in Pakistan [14], but the nonadherence to national and international guidelines and the unavailability of national formularies for the pediatric population could be possible causes of these errors [15]. The incidence of prescribing errors is common in pediatric patients due to age, weight-based dose adjustment, interpatient variability, need of dilution for stock medicines, and altered pharmacokinetic properties of drugs [16]. A study revealed 3–20% errors in prescriptions in the pediatrics inpatient ward [17]. In Pakistan, data of mortality and hospital admissions due to medication prescribing errors is not officially recorded. However, the Pakistan Society of Health System Pharmacists (PSHP) has roughly estimated deaths of 400,000 to 500,000 people (including women and children) annually due to medication errors [18]. Among all the errors, dosing errors are more common in prescription of pediatric patients [19]. Getting an insight about antibiotic prescribing practices and the associated errors among the pediatric population is crucial to improve the current policies and to prevent this population from untoward effects. Therefore, the objective of this study is to compare the antibiotic prescribing pattern and to identify common medication errors in the treatment of ARTIs among pediatric patients with short and long LOS in hospital.

## 2. Methods

### 2.1. Study Design and Settings

A retrospective, cross-sectional study was conducted in five public hospitals (Mayo Hospital, Jinnah Hospital, General Hospital, Services Hospital, and Children’s Hospital) of Lahore, Pakistan. Data were collected between 1 January 2017 and 30 June 2017. The selected hospitals were tertiary-care hospitals providing healthcare services to children in the pediatric department. The characteristics of the selected hospitals are summarized in Supplementary S1.

### 2.2. Study Population and Sample Size

According to hospital records, a total of 249,653 patients (age = 0−9 years) visited the outpatient departments (OPDs) and 86,227 (age = 0−9 year) were admitted during the six-month study period. Among the inpatients, 13,582 were diagnosed with ARTIs. The medical records of all patients aged >9 years were excluded. Participants were included in the study if they were suffering from one ARTI, prescribed with antibiotics after diagnosis of ARTIs, and had no previous medical history of RTIs. The records of 1730 participants who did not meet the inclusion criteria were excluded from the study. Consequently, records of 11,852 inpatients were finally selected for the study (Supplementary S2).

### 2.3. Data Collection

A data collection form was developed consisting of four main parts: (1) characteristics of the patients, (2) diagnosis, (3) recommended antibiotics, and (4) prescribing errors (Supplementary S3). The 2nd edition of the International Classification of Primary Care (ICPC-2) was used to define ARTIs as a clinical diagnosis of LRTIs, acute bronchitis (R 78), pneumonia (R 81), and epiglottitis (R 83). The URTIs included tonsillitis (R 76), whooping cough (R 71), acute bacterial sinusitis (R 75), laryngitis and tracheitis (R 77), and other URTIs, including common cold (R 74) [20]. The Anatomical Therapeutic Chemical (ATC) classification system [21] was used for the coding of antibiotics. The data relating to dose, dosage form, frequency, and duration of prescribed antibiotics were extracted by reviewing patient records according to the criteria (Supplementary S4). Appropriateness of prescribing antibiotics was assessed by critically observing and evaluating each of following indicators: drug selection, dosage form, frequency, prescribed quantity, and duration of therapy (Supplementary S4). The assessment of antibiotic prescribing errors was made by taking into account the clinical judgments, nature and severity of underlying disease, and social factors that may contribute to lengthening the patient’s stay time in hospital. Four investigators were assigned to each selected setting; the principal investigator trained all investigators prior to the survey for the collection and validation of data. During the survey, two investigators filled out the investigational form, and the other two reviewed the medical records of patients. The LOS was evaluated by measuring the difference between the date of admission and the date of discharge [22].

### 2.4. Variables

All the prescribing errors were considered as a dependent variable. The following characteristics were the independent variables and evaluated in the data: gender (1 = male and 0 = female); age (1 = newborn to toddler, 0–3 years; 0 = otherwise), LOS (1 = short, <5 days; 0 = long, ≥5 days); acute respiratory tract infections (1 = LRTI; 0 = URTI).

### 2.5. Definitions

*Prescription errors:* Incorrect or inappropriate drug selection (based upon indications, contraindications, and other factors), dose, route, rate of administration, frequency, or duplication of drugs.
*Wrong frequency*: Drugs prescribed in a frequency other than that recommended by the guidelines.
*Wrong dose*: Prescribed dose greater than or less than 10% of the recommended dose in the guidelines.
*Wrong route*: Prescribed drug administered by a route other than the recommended route mentioned in the guidelines.
*Wrong duration*: Drugs prescribed for a duration other than that recommended in the guidelines.
*Duplicate therapy*: Two or more drugs prescribed from the same class.

### 2.6. Data Analysis

Descriptive statistics were used to present the data. Furthermore, logistic regression analysis was performed to figure out the factors associated with prescribing errors. Results were expressed as odds ratio (OR) accompanied by 95% confidence intervals (95% CI), and a *p*-value < 0.05 was used for statistical significance of differences. Statistical Package for Social Sciences (IBM Corp. Released 2012. IBM SPSS Statistics for Windows Version 21.0. Armonk, NY: IBM Corp.) and Microsoft Excel (MS Office 2010) were used for data analysis.

### 2.7. Ethics Approval 

The ethical approval was obtained from the Pharmacy Research Ethics Committee (PREC) at Akhtar Saeed College of Pharmaceutical Sciences (Reference: 19-2016/PREC, 26 December 2016). Before conducting the study, permission was obtained from hospital administrators. 

## 3. Results

### 3.1. Characteristics of the Patients

Overall, 40.3% (4781) of the patients were aged 0–3 years, and 43.9% (*n* = 5202) were 3–10 kg in weight. Of the participants, 50.9% (*n* = 6033) were prescribed with two antibiotics per prescription, and 53% (*n* = 6285) of patients were admitted for >5 days, i.e., long LOS (Table 1).

### 3.2. Antibiotic Prescribing Practices 

As the present study was conducted in multiple settings, different international guidelines were found to be implemented for different purposes. It was observed that the Current Medical Diagnosis and Treatment (CMDT) [23] and WHO guidelines were used for diagnosis, while the British National Formulary (BNF) [24] was consulted for doses. The American Academy of Family Physicians (AAFP), the American Academy of Pediatrics, the National Institute of Health and Clinical Excellence (NICE) guidelines on RTIs [25], and the American Thoracic Society Consensus Guidelines were consulted for treatment of pneumonia [26]. The IDSP guidelines [27] were considered for assessing the rational use of antibiotics. 

A total of 26,883 antibiotics were prescribed to 11,852 patients. Out of this, 86.9% (*n* = 23,383) of the antibiotics were prescribed to patients suffering from LRTIs, while 13.1% (*n* = 3500) were prescribed to patients with URTIs. The practice of prescribing antibiotics was found to be more frequent among patients who had long LOS (52.9%, *n* = 14,217) compared to those who had short LOS (47.1%, *n* = 12,666). Most of the children were hospitalized due to LRTIs, especially pneumonia (short LOS = 72.9%, long LOS = 90.8%) (Supplementary S5 and S6). Amoxicillin (short LOS = 82.7%, long LOS = 82.8%) and ampicillin (short LOS = 61.8%, long LOS = 61.7%) were the most commonly prescribed antibiotics in pneumonia. Tonsillitis and pharyngitis were the most commonly observed URTIs among inpatients, and azithromycin (short LOS = 31%, long LOS = 47.9%) and amoxicillin (short LOS = 48.1%, long LOS = 30.8%) were the most frequently prescribed antibiotics for this condition (Supplementary S5 and S6).

In ARTIs, antibiotics were more frequently prescribed to patients who had long LOS in hospital compared to those who had short LOS. Penicillins (52.4%, *n* = 14,096), cephalosporins (16.8%, *n* = 4506), and macrolides (8.9%, *n* = 2404) were among the most commonly prescribed classes of antibiotics (Supplementary S7). Amoxicillin, ampicillin, ceftriaxone, and azithromycin were commonly prescribed antibiotics to patients who had long LOS compared to those who had short LOS in the healthcare settings (Table 2).

### 3.3. Antibiotic Prescribing Errors

A total of 4836 (40.8%) records had prescribing errors. Most of the errors were found among patients with LRTIs (82.8%) compared to URTIs (17.2%). 47.2% cases had one error, 21.7% had two errors, while more than three errors were found in 30.9% of those cases in which errors were identified. Wrong dose (especially underdosing), wrong frequency (especially high frequency), and duplicate therapy were the most common errors (Table 3). A large number of errors were found in the records of patients who were hospitalized for long periods of time (45.8%) compared to those who had short LOS (35.2%) (Supplementary S8).

### 3.4. Determinants Associated with Prescribing Errors among Pediatrics Patients

Logistic regression analysis revealed that the odds of prescribing errors decreased by 33% in female patients (OR = 0.6, 95% CI = 0.1–0.9, *p*-value = 0.012) compared to male patients. The odds of prescribing errors were 1.7 times higher in patients who were prescribed with ≥3 antibiotics per prescription (OR = 1.7, 95% CI = 1.1–2.1, *p*-value = 0.020) compared to those who were prescribed with one antibiotic per prescription. In patients with long LOS, the odds of prescribing errors were 12.5 times higher (OR = 12.5, 95% CI = 10.1–17.6, *p*-value < 0.001) compared to patients who had short LOS, while the odds of prescribing errors in patients suffering from URTIs were 2.8 times higher (OR = 2.8, 95% CI = 1.7–3.9, *p*-value < 0.001) compared to those who were suffering from LRTIs (Table 4).

## 4. Discussion

The present study set out to determine the antibiotic prescribing practices among the pediatric population suffering from ARTIs and the medication errors in the prescription records of inpatients with different LOS in hospital. 

### 4.1. Antibiotic Prescribing Practices among Pediatric Patients

Our findings revealed that most of the patients suffering from LRTIs were prescribed with penicillins, while macrolides and cephalosporins were prescribed for URTIs. Among these, amoxicillin, ampicillin, and gentamicin were more frequently prescribed to patients who had long LOS compared to those who had short LOS in the healthcare settings. Evidence from previously published studies suggests that a majority of pediatric patients living in Pakistan are prescribed with cephalosporins after every five hours [28,29]. The unnecessary antibiotic prescribing practices in childhood ARTIs of viral origin is responsible for spreading resistant microbial strains and thereby increasing the disease burden [29]. These findings are comparable with a Jordanian study in which penicillins and cephalosporins were prescribed to a large proportion of the pediatric population [30]. In contrast to developing countries, the rate of antibiotic prescribing among ARTI patients declined from 5.2 to 2.2 prescription per 1000 pediatric patients during the last decade in various states of the UK [31]. In Pakistan, a survey-based study at a national level revealed that ARTIs were the most common indications for prescribing antibiotics and that, in most of the cases, the prescribing practices of many healthcare professionals were irrational [32].

Like the previously published literature [33,34,35], the present study also revealed that antibiotics were more frequently prescribed to patients who had long LOS in hospital compared to those who had short LOS. Most of the ARTIs (such as bronchitis) are of viral origin, and the IDSP guidelines recommend limited use of antibiotics. However, prescribers are compelled to prescribe antibiotics because of their inability to differentiate between viral and bacterial infections; parental demand to prescribe antibiotics; socioeconomic pressures; and to reduce the risk of secondary bacterial infections, duration of cough, and other symptoms [36,37,38]. The noncompliance of these guidelines was also observed in the present study. Macrolides were frequently prescribed in bronchitis, which can be managed through symptomatic treatment. In cases of pneumonia, ampicillin was prescribed as 57 mg/kg/day three times a day instead of 100 mg/kg/day four times a day [39]. These irrational practices are also attributed to the fact that, in Pakistan, pharmacists have a major role in procurement and dispensing of medicines but have limited exposure to the clinical setup and direct patient care. To implement rational antibiotic prescribing practices, the establishment of an antibiotic prescribing surveillance system and continual medical education programs need to be immediately implemented. In case of the pediatric population, clinical pharmacists must participate in parental counseling training programs of healthcare professionals.

### 4.2. Antibiotic Prescribing Errors

The present study revealed that most of the prescribing errors were related to wrong dose (especially underdosing), wrong frequency (especially prescribed greater than the recommended frequency), and duplicate therapy, particularly in the records of patients who had long LOS compared to those who had short LOS. These findings are in line with a retrospective study conducted on Brazilian pediatric patients in which long LOS was found in patients who had been prescribed with antibiotics [40]. Similarly, a prospective study depicted that higher rate of dosing errors in the medication record can threaten patient safety, especially in the pediatric population, because dose calculation is often more complex for these patients [41]. Wrong dose and frequency errors were commonly found among pediatric patients because many of the antibiotics were not readily available in pediatric unit doses, and pediatric doses were therefore calculated from adult unit doses. Antibiotic prescribing errors that are related to their frequent use may not only economically burden the healthcare system by increasing the patient’s LOS in hospital but also subsequently play a crucial role in spreading antibiotic-resistant microbial strains [42]. Nonadherence to national and international guidelines and national formularies for the pediatric population could be the possible causes of these errors [15]. Therefore, hospital formularies and international standard treatment guidelines for pediatrics must be made available in all healthcare settings. To overcome the irrational prescribing of antibiotics, strict diagnostic criteria must be established for ARTIs of bacterial origin. The government must ensure availability of adequate number of hospital and clinical pharmacists in all pediatric public healthcare settings. Their prime responsibilities must include prescription evaluation, calculation of doses, reporting of antibiotic prescribing errors, and counseling of parents about the proper use of antibiotics. Moreover, the Pakistan Pediatric Association (PPA) must take initiatives to develop and implement Pakistan’s own national formulary for pediatrics so that countrywide uniform treatment protocols are established.

The inadequate healthcare system for pediatric patients has led to an increased incidence of prescribing errors and child mortalities. Low state spending on child health, abject poverty, and low literacy rate of parents has indirectly worsened the healthcare system in Pakistan [43]. Therefore, it is recommended that medical and paramedical staff members, along with senior consultants, are increased with the increase in patient load so that each patient gets adequate consultation time, and the risk of prescribing errors are reduced.

### 4.3. Factors Associated with Prescribing Errors

The prescribing errors were less likely to be found in the record of female patients. These findings are in line with previously published studies conducted in different states of Saudi Arabia and Pakistan, where pediatric male gender showed a greater association with prescribing errors [17,44]. Contrary to this, a meta-analysis showed that females were more susceptible to encountering prescribing errors than males [45]. Pakistan is an economically less developed country, where no public child healthcare insurance policy is available. Parents have to bear the health expense of their children on their own, and financial constraints and social norms compel most of the low-income households to prefer medical treatment for male children compared to female children. Due to this reason, male pediatric patients are more likely to be admitted in inpatient settings and are thus at higher risk of encountering prescribing errors than females [46]. 

Similar to previously published studies [47,48,49], LOS was found to be a significant factor for prescribing error. This might be due to the fact patients were prescribed with more number of antibiotics during their prolonged stay in the inpatient departments, which increased the risk of encountering antibiotic prescribing errors. Prescribing errors were more likely to be found in the records of patients suffering from URTIs compared to sufferers of LRTIs. These findings are consistent with previously published studies, where prescribing errors were less commonly found in the prescription records of patients suffering from LRTIs [50,51,52]. This may be attributed to the fact that LRTIs (like pneumonia) are more prevalent in the pediatric population than any URTIs, so specialized medical care is being provided to patients suffering from LRTIs. The prime focus of the available guidelines (such as the IDSP guidelines) is the management of LRTIs because, in most of cases, URTIs are usually mild and self-limiting [53]. Furthermore, statistical analysis revealed significant association between >3 prescribed antibiotics and higher risk of prescribing errors. These findings are consistent with previously published studies, which have shown that the number of antibiotics on prescription is a strong predictor of prescribing error in such a way that the risk of error becomes several folds with the addition of a single antibiotics in prescription [54,55,56]. This may be attributed to various reasons, including diagnostic uncertainties reflecting the physician’s inability to distinguish between viral and bacterial infections, noncompliance of guidelines, poor practices of antimicrobial stewardship (AMS) program, heavy patient load, and unreasonable patient demands on physicians to prescribe antibiotics [57,58,59].

## 5. Strengths and limitations

To the best of our knowledge, this is the first study that deals with antibiotic prescribing errors among pediatric inpatients suffering from ARTIs. These findings will be beneficial to stakeholders in establishing policies and procedures for prescribing antibiotics to children aged 0−9 years. This study emphasized the need for an antibiotic prescribing errors reporting system, a national survey demonstrating rational use of antibiotics among the pediatric population, and the development and implementation of Pakistan own national formulary for pediatrics. 

As with any study, this study also has limitations. First, the results cannot be generalized for Pakistan as a whole because clustering of data at different levels was not taken into account, and multilevel modeling was also not performed to test associated factors. Therefore, antibiotic prescribing practices may vary due to geographic variation of the selected healthcare settings, the prescribing habits of physicians, and the clustering effect. However, the healthcare sector conditions are similar across many regions of the country, and physicians consequently follow the same prescribing practices. We suspect that the findings and the issues raised will be similar in “like” hospitals (i.e., tertiary-care hospitals), so there are relevant learnings for the tertiary sector in Pakistan. Second, as this was a cross-sectional study, it was not possible to see the outcomes of antibiotic prescribing errors, i.e., adverse events and mortalities. Third, the findings of the present study are limited to pediatric patients suffering from only ARTIs because the inclusion of other ARTIs would have complicated the findings. Clinically based longitudinal studies may help to overcome these limitations.

## 6. Conclusions

The present study concluded that penicillins, macrolides, and cephalosporins were frequently prescribed classes of antibiotics in ARTIs, especially in the prescription records of patients who had long LOS. Among these, ampicillin, amoxicillin, azithromycin, and ceftriaxone were the most commonly prescribed agents. Prescription errors like underdosing, high frequency, and duplicate therapy were most commonly found among records of patients who suffered from LRTIs and were admitted for long periods of time in the public hospitals of Lahore. Caution must be exercised in prescribing antibiotics to the pediatric population because antibiotic prescribing errors can be hazardous to lives.

## Figures and Tables

**Table 1:** Characteristics of the patients.

Characteristics		Inpatients (*n* = 11,852)	URTIs * (*n* = 2036)	LRTIs † (*n* = 9816)	*p*-Value
Gender	Male	7481 (63.1)	1219 (59.9)	6262 (63.8)	<0.001
	Female	4371 (36.9)	817 (40.1)	3554 (36.2)	
Age	0–3 years	4781 (40.4)	894 (43.9)	3887 (39.6)	
	4–6 years	4697 (39.6)	662 (32.5)	4035 (41.1)	<0.001
	7–9 years	2374 (20.0)	480 (23.6)	1894 (19.3)	
Weight	3–10 kg	5202 (43.9)	803 (39.4)	4399 (44.8)	
	11–18 kg	3902 (32.9)	851 (41.8)	3051 (31.1)	<0.001
	≥19 kg	2748 (23.2)	382 (18.8)	2366 (24.1)	
Number of antibiotics prescribed per prescription	1	205 (1.8)	98 (4.8)	107 (1.1)	
	2	6433 (54.1)	1109 (54.5)	5324 (54.2)	<0.001
	≥3	5214 (44.1)	829 (40.7)	4385 (44.7)	
LOS	Short (mean = 3.7, range 1–4)	5567 (47.0)	958 (47.1)	4609 (46.9)	
	Long (mean = 6.6, range 5–14)	6285 (53.0)	1078 (52.9)	5207 (53.1)	<0.001

* URTIs = upper respiratory tract infections; † LRTIs = lower respiratory tract infections; LOS = length of stay.

**Table 2:** Prescribing pattern of antibiotics among the study population.

Antibiotics	ATC code	*n* (%)	URTIs			LRTIs		
			Total (3500)	Short LOS (1675)	Long LOS (1826)	Total (23,383)	Short LOS (10,993)	Long LOS (12,392)
Gentamycin	J01GB03	3114 (11.6)	---	---	---	3114 (13.3)	1439 (13.1)	1675 (13.5)
Amoxicillin	J01CA04	7854 (29.2)	584 (16.7)	294 (17.6)	290 (15.9)	7270 (31.1)	3359 (30.6)	3911 (31.6)
Ampicillin	J01CA01	5743 (21.4)	101 (2.9)	38 (2.3)	63 (3.5)	5642 (24.1)	2621 (23.8)	3021 (24.4)
Co-amoxiclav	J01CA04	499 (1.9)	499 (14.3)	222 (13.3)	277 (15.2)	---	---	---
Clarithromycin	J01FA09	540 (2.0)	273 (7.8)	146 (8.7)	127 (6.9)	267 (1.1)	149 (1.4)	118 (0.9)
Azithromycin	J01FA10	1559 (5.8)	333 (9.5)	176 (10.5)	157 (8.6)	1226 (5.2)	589 (5.4)	637 (5.1)
Erythromycin	J01FA01	305 (1.1)	90 (2.6)	43 (2.6)	47 (2.6)	215 (0.9)	120 (1.1)	95 (0.8)
Cefixime	J01DD08	69 (0.2)	69 (1.9)	31 (1.9)	38 (2.1)	---	---	---
Cefuroxime	J01DC02	88 (0.3)	88 (2.5)	38 (2.3)	50 (2.7)	---	---	---
Cefadroxil	J01DB05	103 (0.4)	103 (2.9)	59 (3.5)	44 (2.4)	---	---	---
Cefotaxime	J01DA10	746 (2.8)	207 (5.9)	88 (5.3)	119 (6.5)	539 (2.3)	281 (2.6)	258 (2.1)
Cephalexin	J01DB01	186 (0.7)	186 (5.3)	106 (6.3)	80 (4.4)	---	---	---
Ceftriaxone	J01DA13	3314 (12.3)	517 (14.8)	225 (13.4)	292 (16.0)	2797 (11.9)	1335 (12.2)	1462 (11.8)
Levofloxacin	J01MA12	907 (3.4)	77 (2.2)	34 (2.0)	43 (2.4)	830 (3.5)	383 (3.5)	447 (3.6)
Ciprofloxacin	J01MA02	151 (0.6)	---	---	---	151 (0.6)	79 (0.7)	72 (0.6)
Clindamycin	J01FF01	975 (3.6)	218 (6.2)	109 (6.5)	109 (5.9)	757 (3.2)	360 (3.3)	397 (3.2)
Meropenem	J01DH02	12 (0.1)	12 (0.3)	5 (0.3)	7 (0.4)	---	---	---
Vancomycin	J01XA01	679 (2.5)	104 (2.9)	44 (2.6)	60 (3.3)	575 (2.5)	276 (2.5)	299 (2.4)
Linezolid	J01XX08	39 (0.2)	39 (1.1)	17 (1.1)	22 (1.2)	---	---	---

LOS = length of stay; LRTIs = lower respiratory tract infections; URTIs = upper respiratory tract infections.

**Table 3:** Antibiotic prescribing errors among pediatric patients in Lahore, Pakistan.

Diseases	Patients n (%)	Prescribing Errors								
		Irrational Prescribing n (%)	Wrong Drug n (%)	Wrong Dose n (%)		Wrong Frequency n (%)		Wrong Duration n (%)	Wrong Route n (%)	Duplicate Therapy n (%)
				Over Dose	Underdose	High	Low			
LRTIs										
Pneumonia	8786 (89.5)	3102 (35.3)	404 (13.0)	185 (5.9)	435 (14.0)	341 (10.9)	248 (7.9)	494 (14.9)	433 (13.9)	562 (18.1)
Epiglottitis	731 (7.5)	488 (66.8)	62 (12.7)	42 (8.6)	55 (11.3)	54 (11.1)	39 (7.9)	81 (16.6)	71 (14.6)	84 (17.2)
Bronchitis	299 (3.1)	132 (44.1)	19 (14.4)	8 (6.1)	19 (14.4)	16 (12.1)	8 (6.0)	19 (14.4)	15 (11.4)	28 (21.2)
Total LRTIs	9816 (82.8)	3722 (37.9)	485 (13.0)	235 (6.3)	509 (13.7)	411 (11.0)	295 (7.9)	594(15.9)	519(13.9)	674(18.1)
				744 (19.9)		706 (18.9)				
URTIs										
Tonsillitis and pharyngitis	608 (29.9)	384 (63.1)	48 (12.5)	20 (5.2)	57 (14.8)	42 (10.9)	32 (8.3)	63 (17.0)	57 (14.8)	65 (16.9)
Common cold	123 (6.0)	58 (47.2)	7 (12.1)	6 (10.3)	6 (10.3)	7 (12.1)	3 (5.2)	9 (15.5)	10 (17.2)	10 (17.2)
Whooping cough	171 (8.4)	121 (70.7)	16 (13.2)	7 (5.8)	17 (14.0)	16 (13.2)	7 (5.8)	19 (15.7)	17 (14.0)	22 (18.2)
Acute bacterial sinusitis	577 (28.3)	230 (39.9)	31 (13.5)	13 (5.7)	33 (14.3)	29 (12.6)	14 (6.0)	35 (15.2)	29 (12.6)	46 (20.0)
Bacterial tracheitis	155 (7.6)	128 (82.6)	15 (11.7)	9 (7.0)	17 (13.3)	12 (9.4)	13 (10.2)	22 (17.2)	21 (16.4)	19 (14.8)
Laryngitis and croup	402 (19.8)	193 (48.0)	27 (13.9)	12 (6.2)	26 (13.5)	24 (12.4)	12 (6.2)	29 (15.0)	24 (12.4)	39 (20.2)
Total URTIs	2036 (17.2)	1114 (54.7)	144 (12.9)	67 (6.0)	156 (14.0)	130 (11.7)	81 (7.3)	177 (15.9)	158 (14.2)	201 (18.0)
				223 (20.0)		211 (18.9)				
Overall Total	11,852	4836 (40.8)	629 (13.0)	302 (6.2)	665 (13.8)	541 (11.2)	376 (7.8)	771 (15.9)	677 (13.9)	875 (18.1)
				967 (19.9)		917 (18.9)				

LRTIs = lower respiratory tract infections; URTIs = upper respiratory tract infections; LOS = length of stay.

**Table 4:** Logistic regression analysis of factors associated with prescribing errors.

Characteristics		OR	95% CI	*p*-Value
Gender	Male		------	
	Female	0.6	0.1–0.9	0.012
Age	Newborn to toddler (0–3 years)		------	------
	Preschooler (4–6 years)	0.4	0.01–1.1	0.210
	School aged child (7–9 years)	0.8	0.5–1.4	0.167
Weight	3–10 kg		------	------
	11–18 kg	0.7	0.3–1.2	0.430
	≥19 kg	0.6	0.4–1.9	0.367
Number of antibiotics prescribed per prescription	1		------	------
	2	0.9	0.3–1.6	0.321
	≥3	1.7	1.0–2.1	0.020
LOS	Short (<5 days)		------	------
	Long (≥5 days)	12.5	10.1–17.6	<0.001
ARTIs	URTIs	2.8	1.7–3.9	<0.001
	LRTIs		------	-------

OR: odd ratio; CI: confidence Interval; LOS: length of stay; ARTIs: acute respiratory tract infections; URTIs: upper respiratory tract infections; LRTIs: lower respiratory tract infections.

## Data Availability

The raw data on which conclusions of this manuscript rely is available upon request. Please contact Muhammad Rehan Sarwar at rehansarwaralvi@gmail.com.

## Supplementary Materials

The following are available online at https://www.mdpi.com/1010-660X/55/2/44/s1.
