# Supplementary material for: Antibiotic Prescribing Practices and Errors among Hospitalized Pediatric Patients Suffering from Acute Respiratory Tract Infections: A Multicenter, Cross-Sectional Study in Pakistan
Source: Medicina (Kaunas). 2019 Feb 11;55(2):44. doi: 10.3390/medicina55020044 (PMC6409937; doi:10.3390/medicina55020044)
Supplement: Supplementary file 1 [file medicina-55-00044-s001.pdf]

Supplementary S1: Characteristics of the selected hospitals

Sr. no.	Characteristics	Mayo Hospital	Jinnah Hospital	General Hospital	Services Hospital	Children's Hospital
1	Number of beds	2400	1500	1300	1196	1100
2	Inpatients visit last year	343,114	217,245	134,491	125,868	151,736
3	Prescribers/Medical officers	550	348	300	274	252
4	Pharmacists/Dispensers	30	29	16	14	22
5	Nurses	500	313	271	249	229
6	Other Paramedical staff *	4000	2500	2167	1993	1832

* Other Paramedical staff includes; medical technicians, ward boys, and sweepers.

Supplementary S2: Selection of Study Population

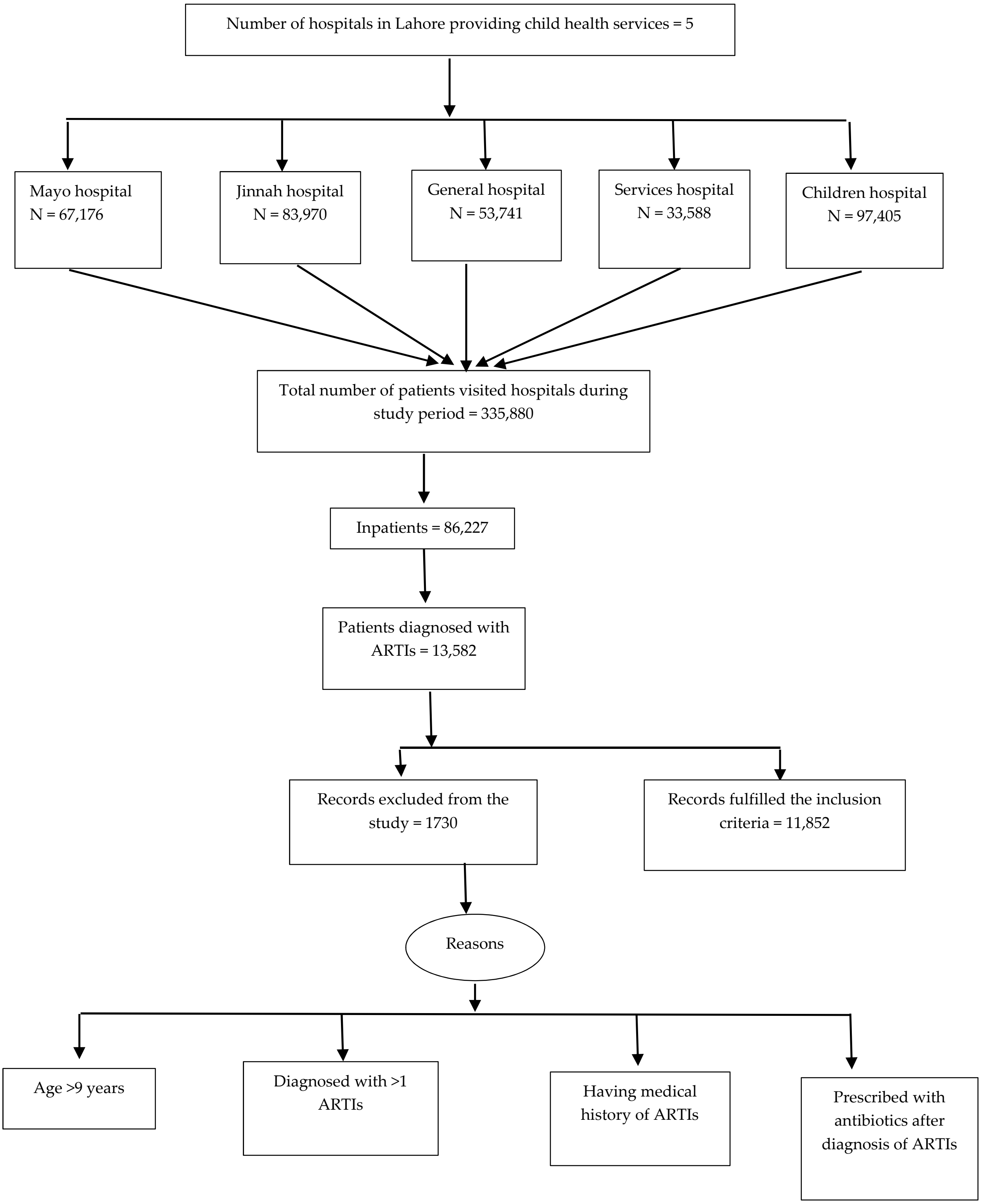

Figure 1: **Selection of Study Population**

Supplementary S3: Questionnaire of the study

Section 1: General information

Name of patient ______________________ Age (years) ____________

Date of hospitalization ______________________ Weight (Kg) ____________

Date of discharge ______________________ Gender ____________

Length of Stay (LOS) (in days) ____________

Height of patient (cm) ______________________

Hospital and ward ____

Name of investigator(s) ____

Name of attending physician ____

Name of clinical pharmacist ____

Section 2: Patient's lab test results and Differential diagnosis

What were the results of patient's lab test reports,

Total leucocyte count (TLC) (cells/µL) ______________________________________

Erythrocytes count (cells/µL) ______________________________________

Platelet count (cells/µL) ______________________________________

Rapid Strep A (RSA) antigen detection test ______________________________________

Throat swab culture test ______________________________________

Respiratory viral panel test ______________________________________

Other microbial/bacterial culture test ______________________________________

What guidelines were being obeyed for diagnosis of ARTIs in the hospital?

____

According to ICPC-2, Patient was suffering from which type of ARTI?

____

Section 3: Patient's medication information

What guidelines were being obeyed or followed in the hospital for prescribing of antibiotics?

____

What antibiotics were prescribed on the basis of differential diagnosis?

Dose ______ Dosage form ______ Route ______ Frequency ______

Duration ______

Antibiotics belong to which class?

Penicillins ☐ Macrolides ☐ Cephalosporins ☐

Fluoroquinolones ☐ Aminoglycosides ☐ Glycopeptide ☐

Lincosamide ☐ Carbapenem ☐ Oxazolidones ☐

Was the prescribed antibiotic therapy in accordance with the guidelines?

☐ Yes ☐ No

In what ways was the prescribed antibiotic regimen differ from the guidelines?

Drug selection ☐ Dose ☐ Route ☐ Frequency ☐

Duration ☐

Section 4: Prescribing errors

Is there any prescribing error found?

Yes ☐ No ☐

How many prescribing errors were found in the record?

☐ One ☐ Two ☐ Three or more

Categorization of prescribing errors

(Check all that apply)

☐ Order written on wrong patient

☐ Order written for wrong drug

- ☐ Drug was inappropriate for indication
- ☐ Patient was allergic to drug
- ☐ Drug-drug or drug-disease interactions

☐ Order written for wrong dose or dose was not adjusted

- ☐ Under dose
- ☐ Over dose

- ☐ Order written for wrong dosing schedule or duration
- ☐ Order written for wrong route
- ☐ Order written for wrong frequency
  - ☐ High frequency
  - ☐ Low frequency
- ☐ Duplication of therapy

Supplementary S4: Definitions of different terms

Terms	Definitions/Criteria
Inpatients	Those who acquired bed in hospital and admitted for >24 hours.
Child	A patient whose age is ≤18 years of age.
Length of stay (LOS)	The duration of a single episode of hospitalization.
Short LOS	If the patient stays is <5 days in the healthcare setting.
Long LOS	If the patient stays is ≥5 days in the healthcare setting.
Wrong drug	Occur when the drug is inappropriate to the patient's medical condition.
Wrong dose	Prescribed dose was ±10% of the recommended dose in the guidelines.
Wrong route	Prescribed drug was administered other than the recommended route by the guidelines.
Wrong frequency	When the prescribed frequency of antibiotics is different from the guidelines.
Wrong duration	When duration of prescribed antibiotics is different from the guidelines e.g., amoxicillin for 7 weeks instead of 7 days.
Duplicate therapy	When multiple antibiotics are prescribed for the same indication without a clear distinction of when one agent should be administered over another

Supplementary S5: Antibiotics prescribed among study population with long length of stay in hospital

Disease	Long LOS* (6285)		Prescribed	Prescribe	Prescribed	Prescribed	Prescribed	N (%)
	Male	Female	Antibiotics	d Dose	Route	Duration	Frequency	
LRTIs† = 5207 (82.9%), Prescribed antibiotics = 12,392 (87.2%)								
Pneumonia 4726 (90.8)	3101 (65.6)	1625 (34.4)	Amoxicillin	46 mg/kg	Oral	5 days	BID	3911 (82.8)
			Ampicillin	59.5 mg/kg	IM/ IV	5 days	TID	2918 (61.7)
			Gentamicin	9.1 mg/kg	IM/IV	5 days	QD	1675 (35.4)
			Ceftriaxone	51 mg/kg	IV	5 days	QD	1112 (23.5)
			Azithromycin	7.4 mg/kg	Oral	5.5 days	BID	534 (11.3)
			Levofloxacin	8.1 mg/kg	IV	5days	QD	447 (9.5)
			Clindamycin	14 mg/kg	IV	7 days	TID	316 (6.7)
			Vancomycin	17.5 mg/kg	IV	5days	TID	132 (2.8)
							QID	87 (1.8)
Epiglottitis 349 (6.7)	227 (65.0)	122 (34.9)	Cefotaxime	45 mg/kg	IV	5days	QID	92 (26.4)
							TID	55 (15.8)
					IM		QID	73 (20.9)
							TID	38 (10.9)
			Ceftriaxone	75 mg/kg	IV/IM	5 days	QD	148 (42.4)
					IV/IM		BID	61 (17.5)
				100 mg/kg	IV/IM		QD	97 (27.8)
					IV/IM		BID	44 (12.6)
			Ampicillin	200 mg/kg	IV	6 days	QID	103 (29.5)
			Ciprofloxacin	200 mg/kg	IV	5 days	QID	72 (20.6)
			Clindamycin	25 mg/kg	IV	10days	QID	81 (23.2)
			Vancomycin	43.5 mg/kg	IV	5 days	TID	36 (10.3)
					IV		QID	44 (12.6)
Bronchitis 132 (2.5)	85 (64.4)	47 (35.6)	Azithromycin	29.1 mg/kg	Oral	5 days	BID	103 (78.0)
			Clarithromycin	9.5 mg/kg	Oral	7 days	BID	118 (89.4)
			Erythromycin	50 mg/kg	Oral	14 days	QID	95 (71.9)
URTIs‡ = 1078 (17.2%), Prescribed antibiotics = 1826 (12.8%)								

Tonsillitis and Pharyngitis 263 (5.1)	121 (46.0)	142 (54)	Amoxicillin	58 mg/kg	Oral	10 days	QD	126 (47.9)
			Cephalexin	22 mg/kg 51.5 mg/kg	Oral	10 days	BID	80 (30.4)
			Cefadroxil	33 mg/kg	Oral	10 days	QD	44 (16.7)
			Clindamycin	7.4 mg/kg	Oral	10 days	TID	50 (19.0)
			Azithromycin	14 mg/kg	Oral	5 days	QD	81 (30.8)
			Clarithromycin	7.5 mg/kg 17 mg/kg	Oral	10 days	BID	69 (26.2)
Common cold 72 (1.4)	35 (48.6)	37 (51.4)	Amoxicillin	90.3 mg/kg	Oral	10 days	BID	26 (36.1)
			Co-amoxiclav	87 mg/kg	Oral	7 days	BID	22 (30.6)
			Cefuroxime	34.6 mg/kg	Oral	10 days	BID	17 (23.6)
			Ceftriaxone	52 mg/kg	IM/IV	10days	BID	15 (20.8)
			Clindamycin	40.7 mg/kg	IV	7days	TID	12 (16.7)
			Erythromycin	43 mg/kg	Oral	10days	TID	10 (13.9)
			Azithromycin	12 mg/kg	Oral	5 day	BID	8 (11.1)
				7.6 mg/kg	Oral	5days	BID	9 (12.5)
			Clarithromycin	13 mg/kg	Oral	10days	BID	6 (8.3)
Whooping cough 103 (1.9)	60 (58.3)	43 (41.7)	Azithromycin	8 mg/kg	Oral	5days	QD	59 (57.3)
			Clarithromycin	6.5 mg/kg	Oral	7days	BID	53 (51.5)
			Erythromycin	50 mg/kg	Oral	14days	QID	37 (35.9)
Acute bacterial Sinusitis 321 (6.2)	193 (60.1)	128 (39.9)	Amoxicillin	90 mg/kg	Oral	7 days	BID	138 (42.9)
			Co-amoxiclav	80 mg/kg	Oral	5 days	BID	50 (15.6)
			Cefixime	6 mg/kg	Oral	10 days	QD	38 (11.8)
			Cefuroxime	35 mg/kg	Oral	5 days	BID	33 (10.3)
			Linezolid	30 mg/kg	Oral	7 days	BID	22 (6.9)
			Levofloxacin	15 mg/kg	Oral	5 days	BID	43 (13.4)
			Cefotaxime	200 mg/kg	IV	7 days	TID	88 (27.4)
			Ceftriaxone	100 mg/kg	IV	7days	BID	66 (20.6)
			Clindamycin	40 mg/kg	IV	5 days	TID	37 (11.5)
			Vancomycin	60 mg/kg	IV	5 days	TID	37 (11.5)
Bacterial Tracheitis 97 (1.9)	54 (55.7)	43 (44.3)	Ampicillin	200 mg/kg	IV	7 days	QD	63 (64.9)
			Ceftriaxone	100 mg/kg	IV	5 days	QD	35 (36.1)
			Cefotaxime	200 mg/kg	IV	5 days	BID	31 (31.9)
			Vancomycin	60 mg/kg	IV	7days	BID	23 (23.7)
			Clindamycin	42 mg/kg	IV	7days	BID	10 (10.3)

			**Meropenem**	**120 mg/kg**	**IV**	**5 days**	**BID**	**7 (7.2)**
**Laryngitis and Croup 222 (4.3)**	**136 (61.3)**	**86 (38.7)**	**Ceftriaxone**	**80 mg/kg**	**Oral**	**5 days**	**QD**	**176 (79.3)**
			**Co-amoxiclav**	**47 mg/kg**	**Oral**	**7 days**	**BID**	**205 (92.3)**

* ATC = Anatomical Therapeutic Chemical; † URTIs = Upper respiratory tract infections, ‡ LRTIs = Lower respiratory tract infection, LOS = Length of stay

Supplementary S6: Antibiotics prescribed among study population with short length of stay in hospital

Disease	Short LOS* (5567)		Prescribed Antibiotics	Prescribed Dose	Prescribed Route	Prescribed Duration	Prescribed Frequency	N (%)
	Male	Female						
LRTIs† = 4609 (82.8%), Prescribed antibiotics = 10,993 (86.8%)								
Pneumonia 4060 (72.9)	2577 (63.5)	1483 (36.5)	Amoxicillin	40 mg/kg	Oral	3 days	BID	3359 (82.7)
			Ampicillin	51.8 mg/kg	IM/IV	4 days	TID	2509 (61.8)
			Gentamicin	7 mg/kg	IM/IV	3 days	QD	1439 (35.4)
			Ceftriaxone	43.2 mg/kg	IV	3 days	QD	955 (23.5)
			Azithromycin	5.4 mg/kg	Oral	3 days	BID	459 (11.3)
			Levofloxacin	6.1 mg/kg	IV	4.5 days	QD	383 (9.4)
			Clindamycin	10.5 mg/kg	IV	4 days	TID	271 (6.7)
			Vancomycin	15.5 mg/kg	IV	3 days	TID	113 (2.8)
							QID	75 (1.9)
Epiglottitis 382 (6.9)	195 (51.1)	187 (48.9)	Cefotaxime	25.4 mg/kg	IV/IM	3 days	QID	98 (25.7)
							TID	60 (15.7)
							QID	81 (21.2)
							TID	42 (10.9)
			Ceftriaxone	50.2 mg/kg	IV/IM	3.5 days	QD	160 (41.9)
					IV/IM		BID	66 (17.3)
				80.3 mg/kg	IV/IM		QD	106 (27.8)
					IV/IM		BID	48 (12.6)
			Ampicillin	120 mg/kg	IV	3 days	QID	114 (29.8)
			Ciprofloxacin	150 mg/kg	IV	3 days	QID	79 (20.7)
			Clindamycin	15.5 mg/kg	IV	4 days	QID	89 (23.3)
			Vancomycin	40.5 mg/kg	IV	3 days	TID	40 (10.5)
					IV		QID	48 (12.6)
Bronchitis 167 (2.9)	77 (46.1)	90 (53.9)	Azithromycin	25.1 mg/kg	Oral	3 days	BID	130 (77.8)
			Clarithromycin	9.5 mg/kg	Oral	4 days	BID	149 (89.2)
			Erythromycin	40.3 mg/kg	Oral	4.5 days	QID	120 (71.9)
URTIs‡ = 958 (17.2%), Prescribed antibiotics = 1675 (13.2%)								
Tonsillitis and Pharyngitis 345 (6.2)	249 (72.2)	96 (27.8)	Amoxicillin	53 mg/kg	Oral	4.5 days	QD	166 (48.1)
			Cephalexin	20.3 mg/kg<br>41.4 mg/kg	Oral	4 days	BID	106 (30.7)
			Cefadroxil	33 mg/kg	Oral	4.5 days	QD	59 (17.1)
			Clindamycin	6.4 mg/kg	Oral	4 days	TID	65 (18.8)

			Azithromycin	11.5 mg/kg	Oral	3 days	QD	107 (31.0)
			Clarithromycin	5.5 mg/kg<br>14 mg/kg	Oral	4.5 days	BID	91 (26.4)
Common cold<br>51 (0.9)	17<br>(33.3)	34<br>(66.7)	Amoxicillin	80.6 mg/kg	Oral	4 days	BID	18 (35.3)
			Co-amoxiclav	80.1 mg/kg	Oral	4 days	BID	16 (31.4)
			Cefuroxime	31 mg/kg	Oral	4.5 days	BID	12 (23.5)
			Ceftriaxone	39.7 mg/kg	IM<br>IV	4 days	BID	11 (21.6)
			Clindamycin	30.3 mg/kg	IV	3.5 days	TID	9 (17.7)
			Erythromycin	40.1 mg/kg	Oral	3 days	TID	7 (13.7)
			Azithromycin	11.3 mg/kg<br>6 mg/kg	Oral<br>Oral	1 day<br>4 days	BID<br>BID	5 (9.8)<br>6 (11.8)
			Clarithromycin	11.1 mg/kg	Oral	4 days	BID	4 (7.8)
Whooping cough 68 (1.2)	29<br>(42.7)	39<br>(57.4)	Azithromycin	7 mg/kg	Oral	3 days	QD	58 (85.3)
			Clarithromycin	6.5 mg/kg	Oral	3 days	BID	51 (75.0)
			Erythromycin	40 mg/kg	Oral	4 days	QID	36 (52.9)
Acute bacterial Sinusitis<br>256 (4.6)	177<br>(69.1)	79<br>(30.9)	Amoxicillin	40.9 mg/kg	Oral	3 days	BID	110 (42.9)
			Co-amoxiclav	78.5 mg/kg	Oral	3 days	BID	39 (15.2)
			Cefixime	6 mg/kg	Oral	4 days	QD	31 (12.1)
			Cefuroxime	30.4 mg/kg	Oral	2.5 days	BID	26 (10.2)
			Linezolid	20.3 mg/kg	Oral	3 days	BID	17 (6.6)
			Levofloxacin	15 mg/kg	Oral	3.5 days	BID	34 (13.3)
			Cefotaxime	150 mg/kg	IV	4 days	TID	71 (27.7)
			Ceftriaxone	50 mg/kg	IV	3.5days	BID	53 (20.7)
			Clindamycin	20 mg/kg	IV	3 days	TID	29 (11.3)
			Vancomycin	40 mg/kg	IV	3 days	TID	30 (11.7)
Bacterial Tracheitis<br>58 (1.0)	26<br>(44.8)	32<br>(55.2)	Ampicillin	150 mg/kg	IV	4 days	QD	38 (65.5)
			Ceftriaxone	50 mg/kg	IV	3.5 days	QD	18 (31.0)
			Cefotaxime	150 mg/kg	IV	3 days	BID	17 (29.3)
			Vancomycin	40 mg/kg	IV	3.5days	BID	14 (24.1)
			Clindamycin	38.4 mg/kg	IV	3 days	BID	6 (10.4)
			Meropenem	60 mg/kg	IV	3 days	BID	5 (8.6)
Laryngitis and Croup<br>180 (3.2)	122<br>(67.8)	58<br>(32.2)	Ceftriaxone	20 mg/kg	Oral	3.5 days	QD	143 (79.4)
			Co-amoxiclav	41.5 mg/kg	Oral	3 days	BID	167 (92.8)

Supplementary S7: Pharmacological classes of antibiotics prescribed among study population

Class	ATC code*	N (%)	URTIs†			LRTIs‡		
			Total (3500)	Short LOS (1675)	Long LOS (1825)	Total (23,383)	Short LOS (10,991)	Long LOS (12,392)
Aminoglycosides	J01G	3114 (11.6)	---	---	---	3114 (13.3)	1439 (13.1)	1675 (13.5)
Penicillins	J01C	14,096 (52.4)	1184 (33.8)	554 (33.1)	630 (34.5)	12,912 (55.2)	5980 (54.4)	6932 (55.9)
Macrolides	J01FA	2404 (8.9)	696 (19.9)	365 (21.8)	331(18.3)	1708 (7.3)	858 (7.8)	850 (6.9)
Cephalosporins	J01D	4506 (16.8)	1170 (33.4)	547 (32.7)	623 (34.1)	3336 (14.3)	1616 (14.7)	1720 (13.9)
Fluoroquinolones	J01M	1058 (3.9)	77 (2.2)	34 (2.0)	43 (2.4)	981 (4.2)	462 (4.2)	519 (4.2)
Lincosamide	J01FF	975 (3.6)	218 (6.2)	109 (6.5)	109 (5.9)	757 (3.2)	360 (3.3)	397 (3.2)
Carbapenem	J01DH	12 (0.1)	12 (0.3)	5 (0.3)	7 (0.4)	---	---	---
Glycopeptide	J01XA	679 (2.5)	104 (2.9)	44 (2.6)	60 (3.3)	575 (2.5)	276 (2.5)	299 (2.4)
Oxazolidones	J01XX	39 (0.2)	39 (1.1)	17 (1.0)	22 (1.2)	---	---	---

* ATC = Anatomical Therapeutic Chemical; † URTIs = Upper respiratory tract infections, ‡ LRTIs = Lower respiratory tract infections; LOS = Length of stay

Supplementary S8: Antibiotic prescribing errors among patients with short and long stay in hospital

Diseases	Patients N (%)	Prescribing errors								
		Irrational prescribing N (%)	Wrong drug N (%)	Wrong dose N (%)		Wrong Frequency N (%)		Wrong duration N (%)	Wrong Route N (%)	Duplicate therapy N (%)
				Over dose	Under dose	High	Low			
				LRTIs*						
Pneumonia	Short LOS 4060 (46.2)	1296 (31.9)	145 (11.2)	38 (2.9)	201 (15.5)	132 (10.2)	109 (8.4)	287 (22.2)	162 (12.5)	222 (17.1)
	Long LOS 4726 (53.8)	1806 (38.2)	259 (14.3)	147 (8.1)	234 (12.9)	209 (11.6)	139 (7.7)	207 (11.5)	271 (15.0)	340 (18.8)
	Total 8786 (89.5)	3102 (35.3)	404 (13.0)	185 (5.9)	435 (14.0)	341 (10.9)	248 (7.9)	494 (14.9)	433 (13.9)	562 (18.1)
Epiglottitis	Short LOS 382 (52.3)	197 (51.6)	28 (14.2)	11 (5.6)	19 (9.6)	23 (11.7)	17 (8.6)	34 (17.3)	39 (19.8)	26 (13.2)
	Long LOS 349 (47.7)	291 (83.4)	34 (11.7)	31 (10.5)	36 (12.2)	31 (10.5)	22 (7.5)	47 (15.9)	32 (10.9)	58 (19.7)
	Total 731 (7.5)	488 (66.8)	62 (12.7)	42 (8.6)	55 (11.3)	54 (11.1)	39 (7.9)	81 (16.6)	71 (14.6)	84 (17.2)
Bronchitis	Short LOS 167 (55.9)	45 (26.9)	6 (13.3)	2 (4.4)	8 (17.8)	3 (6.7)	1 (2.2)	9 (20.0)	5 (11.1)	11 (24.4)
	Long LOS 132 (44.1)	87 (65.9)	13 (14.9)	6 (6.9)	11 (12.6)	13 (14.9)	7 (8.1)	10 (11.5)	10 (11.5)	17 (19.5)
	Total 299 (3.1)	132 (44.1)	19 (14.4)	8 (6.1)	19 (14.4)	16 (12.1)	8 (6.0)	19 (14.4)	15 (11.4)	28 (21.2)
Total LRTIs	Short LOS 4609 (46.9)	1538 (33.4)	179 (11.6)	51 (3.3)	228 (14.8)	158 (10.3)	127 (8.3)	330 (21.5)	206 (13.4)	259 (16.8)
	Long LOS 5207 (53.1)	2184 (41.9)	306 (14.0)	184 (8.4)	281 (12.9)	253 (11.6)	168 (7.7)	264 (12.1)	313 (14.3)	415 (19.0)
	Total 9816 (82.8)	3722 (37.9)	485 (13.0)	235 (6.3)	509 (13.7)	411 (11.0)	295 (7.9)	594(15.9)	519(13.9)	674(18.1)
				744 (19.9)		706 (18.9)				
				URTIs†						
Tonsillitis and Pharyngitis	Short LOS 345 (56.7)	144 (41.7)	15 (10.4)	3 (2.1)	26 (18.1)	17 (11.8)	11 (7.6)	24 (16.7)	21 (14.6)	27 (18.8)
	Long LOS 263 (43.3)	240 (91.3)	33 (13.8)	17 (7.1)	31 (12.9)	25 (10.4)	21 (8.8)	39 (16.3)	36 (15.0)	38 (15.8)
	Total 608 (29.9)	384 (63.1)	48 (12.5)	20 (5.2)	57 (14.8)	42 (10.9)	32 (8.3)	63 (17.0)	57 (14.8)	65 (16.9)
Common cold	Short LOS 51 (41.5)	19 (37.3)	2 (10.5)	1 (5.3)	1 (5.3)	3 (15.8)	2 (10.5)	4 (21.1)	5 (26.3)	1 (5.3)
	Long LOS 72 (58.5)	39 (54.2)	5 (12.8)	5 (12.8)	5 (12.8)	4 (10.3)	1 (2.6)	5 (12.8)	5 (12.8)	9 (23.1)
	Total 123 (6.0)	58 (47.2)	7 (12.1)	6 (10.3)	6 (10.3)	7 (12.1)	3 (5.2)	9 (15.5)	10 (17.2)	10 (17.2)
Whooping cough	Short LOS 68 (39.8)	51 (75.0)	7 (13.7)	3 (5.9)	8 (15.7)	7 (13.7)	2 (3.9)	10 (19.6)	5 (9.8)	9 (17.6)
	Long LOS 103 (60.2)	70 (67.9)	9 (12.9)	4 (5.7)	9 (12.9)	9 (12.9)	5 (7.1)	9 (12.9)	12 (17.1)	13 (18.6)
	Total 171 (8.4)	121 (70.7)	16 (13.2)	7 (5.8)	17 (14.0)	16 (13.2)	7 (5.8)	19 (15.7)	17 (14.0)	22 (18.2)
Acute Bacterial Sinusitis	Short LOS 256 (44.4)	86 (33.6)	11 (12.8)	2 (2.3)	9 (10.5)	13 (15.1)	4 (4.7)	16 (18.6)	10 (11.6)	21 (24.4)
	Long LOS 321 (55.6)	144 (44.9)	20 (13.9)	11 (7.6)	24 (16.7)	16 (11.1)	10 (6.9)	19 (13.2)	19 (13.2)	25 (17.4)
	Total 577 (28.3)	230 (39.9)	31 (13.5)	13 (5.7)	33 (14.3)	29 (12.6)	14 (6.0)	35 (15.2)	29 (12.6)	46 (20.0)

Bacterial Tracheitis	Short LOS 58 (37.4)	51 (87.9)	4 (7.8)	2 (3.9)	10 (19.6)	3 (5.9)	4 (7.8)	9 (17.6)	11 (21.6)	8 (15.7)
	Long LOS 97 (62.6)	77 (79.4)	11 (14.3)	7 (9.1)	7 (9.1)	9 (11.7)	9 (11.7)	13 (16.9)	10 (12.9)	11 (14.3)
	Total 155 (7.6)	128 (82.6)	15 (11.7)	9 (7.0)	17 (13.3)	12 (9.4)	13 (10.2)	22 (17.2)	21 (16.4)	19 (14.8)
Laryngitis and Croup	Short LOS 180 (44.8)	71 (39.4)	6 (8.5)	2 (2.8)	13 (18.3)	10 (14.1)	3 (4.2)	14 (19.7)	8 (11.3)	15 (21.1)
	Long LOS 222 (55.2)	122 (54.9)	21 (17.2)	10 (8.2)	13 (10.7)	14 (11.5)	9 (7.4)	15 (12.3)	16 (13.1)	24 (19.7)
	Total 402 (19.8)	193 (48.0)	27 (13.9)	12 (6.2)	26 (13.5)	24 (12.4)	12 (6.2)	29 (15.0)	24 (12.4)	39 (20.2)
Total URTIs	Short LOS 958 (47.1)	422 (44.1)	45 (10.7)	13 (3.1)	67 (15.9)	53 (12.6)	26 (6.2)	77 (18.3)	60 (14.2)	81 (19.2)
	Long LOS 1078 (52.9)	692 (64.2)	99 (14.3)	54 (7.8)	89 (12.9)	77 (11.1)	55 (7.9)	100 (14.5)	98 (14.2)	120 (17.3)
	Total 2036 (17.2)	1114 (54.7)	144 (12.9)	67 (6.0)	156 (14.0)	130 (11.7)	81 (7.3)	177 (15.9)	158 (14.2)	201 (18.0)
				223 (20.0)		211 (18.9)				
Overall Total	Short LOS 5567 (46.9)	1960 (35.2)	224 (11.4)	64 (3.3)	295 (15.1)	211 (10.8)	153 (7.8)	407 (20.8)	266 (13.6)	340 (17.4)
	Long LOS 6285 (53.1)	2876 (45.8)	405 (14.1)	238 (8.3)	370 (12.9)	330 (11.5)	223 (7.8)	364 (12.7)	411 (14.3)	535 (18.6)
	Total 11,852	4836 (40.8)	629 (13.0)	302 (6.2)	665 (13.8)	541 (11.2)	376 (7.8)	771 (15.9)	677 (13.9)	875 (18.1)
				967 (19.9)		917 (18.9)				
